# Microscale acoustic metamaterials as conformal sonotransparent skull prostheses

**DOI:** 10.21203/rs.3.rs-2743580/v1

**Published:** 2023-05-10

**Authors:** Gunho Kim, Claire Rabut, Bill Ling, Mikhail Shapiro, Chiara Daraio

**Affiliations:** California Institute of Technology; California Institute of Technology; California Institute of Technology; California Institute of Technology; California Institute of Technology

## Abstract

Functional ultrasound imaging enables sensitive, high-resolution imaging of neural activity in freely behaving animals and human patients. However, the skull acts as an aberrating and absorbing layer for sound waves, leading to most functional ultrasound experiments being conducted after skull removal. In pre-clinical settings, craniotomies are often covered with a polymethylpentene film, which offers limited longitudinal imaging, due to the film’s poor conformability, and limited mechanical protection, due to the film’s low stiffness. Here, we introduce a skull replacement consisting of a microstructured, conformal acoustic window based on mechanical metamaterials, designed to offer high stiffness-to-density ratio and sonotransparency. We test the acoustic window in vivo, via terminal and survival experiments on small animals. Long-term biocompatibility and lasting signal sensitivity are demonstrated over a long period of time (> 4 months) by conducting ultrasound imaging in mouse models implanted with the metamaterial skull prosthesis.

## Introduction

Functional ultrasound imaging (fUSI), the ultrasound analogue of functional magnetic resonance imaging (fMRI), enables the imaging of whole-brain activity with high spatio-temporal resolution and high sensitivity^1,2^. Based on the power Doppler technique^3^, fUSI records brain dynamics by measuring the variation of cerebral blood volume, indirectly coupled to cerebral activity through the neurovascular coupling. fUSI has been used in many different animal models from rodent^4,5^ to primate^6^ and in humans^7,8^, and it can easily be combined with other brain recording technology such as optical^9^ or electrical modalities^10^. Moreover, fUSI can easily be adapted for awake head-fixed or freely-moving animals^11^ and it is suitable for pharmacological studies using functional connectivity as a readout^12^.

To ensure high sensitivity to smallest blood volume variations, fUSI relies on high frequencies (typically between 5 MHz and 15 MHz), which are sensitive to bone’s attenuation and aberration^13^. This is different from low-frequency focused ultrasounds (typically between 0.2 MHz to 1 MHz) used for therapeutic applications, where transcranial procedures are possible^14^. As a result, most fUSI applications require circumventing the skull, through open craniotomy^4,6^ or thinned skull procedures^15^. For chronic studies, the skull can be replaced with an acoustically transparent bio-polymeric cranial window, to preserve the integrity of brain tissues over time^10,16^. The literature has extensively reported the use of polymethylpentene (PMP) sheets to replace the skull for ultrasound imaging experiments (see references in Table S1). However, PMP sheets are thin, not conformal and have a low Young’s modulus (< 2 GPa)^17^, which make them unsuitable to serve as a mechanical protection against external stresses.

An improved skull-replacement prosthesis should: (i) be tailorable to match the shape of individual anatomical features, (ii) be stiff to serve as protection for the brain, and (iii) be sonotransparent. Essentially, it is desirable to create conformal acoustic windows, with quasi-static mechanical properties matching the skull and with acoustic impedance matching the brain.

Mechanical metamaterials (MMs) are rationally designed materials that derive their properties from the selection of their constitutive materials and from the geometry of their micro- and meso-structures. MMs have been shown to exhibit unprecedented mechanical properties, in both static and dynamic loading regimes. For example, MMs can have very high stiffness and strength at low density^18–20^, or are capable of manipulating elastic and acoustic waves beyond naturally defined limits^21–23^. Acoustic metamaterials (AMMs) are the subset of MMs aimed at manipulating acoustic waves, capable of achieving selective transmission^24–35^, cloaking^36,37^, or focusing and lensing^38,39^. AMMs can achieve near perfect transmission via resonance^25^, zero or negative density^26,27^, narrow apertures^28,29^, impedance matching^30,31^, and overcome the presence of aberrating layers^32–35^. However, most of the proposed solutions only work within a narrow frequency bandwidth, which limits their applications for broader use^24,25,28,29, 32–35^. Earlier works demonstrated the use of MMs to image through a stiff and lossy barrier^33,34^, however, these AMMs do not conform to a real skull geometry and do not account for the irregularities and inhomogeneities of bone. As such, existing designs are not readily applicable to solve in vivo problems.

### Mechanical metamaterials as a conformal cranial windows

Here, we focus on the realisation of MMs that can be implanted as custom skull replacements (or “metaskulls”, Fig. 1a,b). To achieve high effective stiffness along the direction normal to the skull’s surface as protection for the brain, we design a metaskull’s microstructure as a hexagonal, honeycomb lattice with perforated panels (Fig. 1a). Honeycomb plate lattices are known to reach the highest stiffness values, at constant density for two-phase materials, loaded along the vertical direction (topping the Hashin-Shtrikman bound)^40^. To ensure acoustic transparency around 15 MHz, to meet the requirements for fUSI in small animals^1^, we design structural features in the micrometre scale and fabricate the metaskulls using 2-photon polymerization (2PP) (Fig. 1c).

The metaskulls are composed of polymerized IP-S, which is an acrylic polymer cured from its viscous liquid photoresist state, suitable for fabricating biocompatible microscale materials with intricate inner structures^41^. The unit cells of the honeycomb microlattices consist of vertical panels (Fig. 1c) that enclose an air filled cavity, to match the acoustic impedance of biological tissue (*Z*_*tissue*_ ~ 1.5 MRayl). To ensure that uncured photoresist trapped inside the cavities can be removed after fabrication, we include horizontal drainage holes (5 µm in diameter) on all vertical panels.

To numerically evaluate the quasi-static and dynamic mechanical properties of the metaskulls, we implement finite element (FE) models, which we validate with experiments. We also conduct in vivo tests in mice, to evaluate the brain imaging quality through the metaskulls of varying thickness, by measuring both the total intensity and signal-to-noise ratio (SNR) of the signal. To demonstrate the long-term stability of the metaskulls for brain imaging, we perform longitudinal experiments in vivo via Doppler ultrasound imaging with visual stimulation.

### Stiffness of metaskulls for mechanical protection

**Figure 2:**
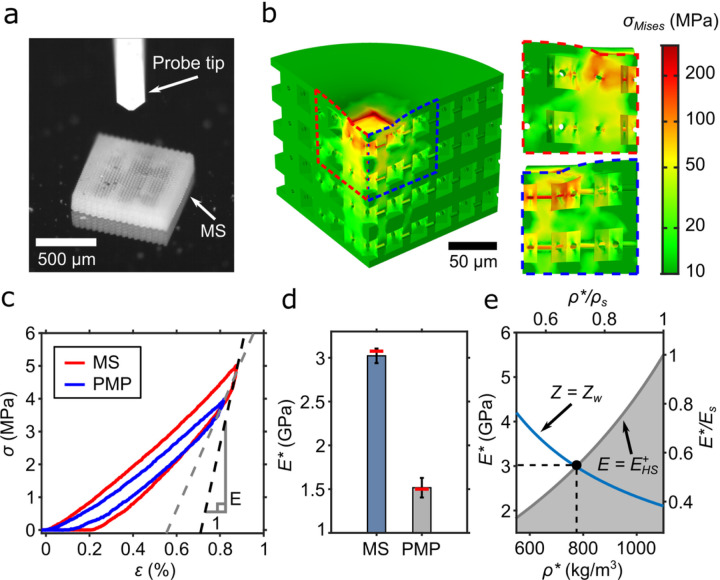
Quasi-static mechanical characterization of a metaskull (MS). a, Optical microscopy image of a metaskull section used for compression testing, showing the probe tip of the measurement system. b, Von Mises stress distribution calculated with the FE model compressed with a 50 µm X 50 µm square-faced probe tip. Only a quarter of the model and its side views are shown. c, Experimental stress-strain curves showing a single loading-unloading cycle of the metaskull and of a PMP film under indentation. Dashed lines indicate the slopes at the onset of the unloading curve, which are used to calculate the effective Young’s moduli. d, The average and standard deviation of the Young’s modulus obtained from experiments, for both the honeycomb plate-lattices and a PMP film. Red lines indicate the predicted values from the numerical simulations (MS) or the known material properties (PMP). e, Theoretical upper bound for the modulus (E+HS) of a two-phase, isotropic material (gray line) crossed by an isoline matching the acoustic impedance of the brain (blue line), both as a function of the Young’s modulus and density of the constituent solid. Black point indicates the properties of the metaskull: (EMS = 3.02 GPa, *ρ*MS = 775.6 kg/m3). The acoustic impedance of water (Zw) is constant at 1.48 MRayl and follows a relation, Z=√*ρ*E. The normalised Young’s modulus (E*/Es) and relative density (ρ*/*ρ*s) are normalised with the Young’s modulus, 5.5 GPa, and density, 1100 kg/m3, of IP-S, respectively.

We numerically calculate the effective mechanical response of a metaskull’s microstructure under compression using a linear elastic model, with a commercial FE software (COMSOL Multiphysics^®^) (see SI for more information). The FE model is configured to reproduce the experimental setup (FemtoTools AG, Fig. 2a). Numerical simulations are used to compute the deformation and stress distribution of a 4-unit-cell-thick, finite-sized honeycomb lattice (Fig. 2b). To minimise boundary effects, the lattice model is designed to be much larger than the compression tip (Fig. S2). The effective compressive Young’s modulus of the honeycomb lattice evaluated from numerical simulation is E* = 3.08 GPa (see Methods). In comparison, the elastic modulus of the 125-µm-thick PMP is *E* = 1.5 GPa^17^. As expected, the von Mises stress distribution of the model shows that the stress and deformations are concentrated below the compressed region, especially around the horizontal drainage holes. The presence of the horizontal holes decreases the effective stiffness of the honeycomb plate-lattices by 12.4% (Fig. S4).

The mean effective compressive modulus measured from independent compression tests of the 3D-printed honeycomb plate-lattices is 3.02 GPa ± 83.4 MPa (standard deviation), which is in good agreement with the numerical prediction (3.08 GPa) (Fig. 2d). As a reference, we also measured the compressive response of a PMP film, from which we extracted a Young’s modulus of 1.52 GPa ± 112.1 MPa, demonstrating that the mechanical performance of the metaskull is superior to conventional materials used in practice.The dimensions of the honeycomb plate-lattice unit cells were selected so that the metaskulls can be impedance-matched to biological tissue, while preserving the highest possible quasi-static stiffness. As expected, the stiffness of the honeycomb metamaterials lies on the edge of the theoretical limit, which is the Hashin-Shtrikman upper bound for two phase materials^42^ (Fig. 2e).

### Acoustic characteristics of metaskulls shows sonotransparency

**Figure 3:**
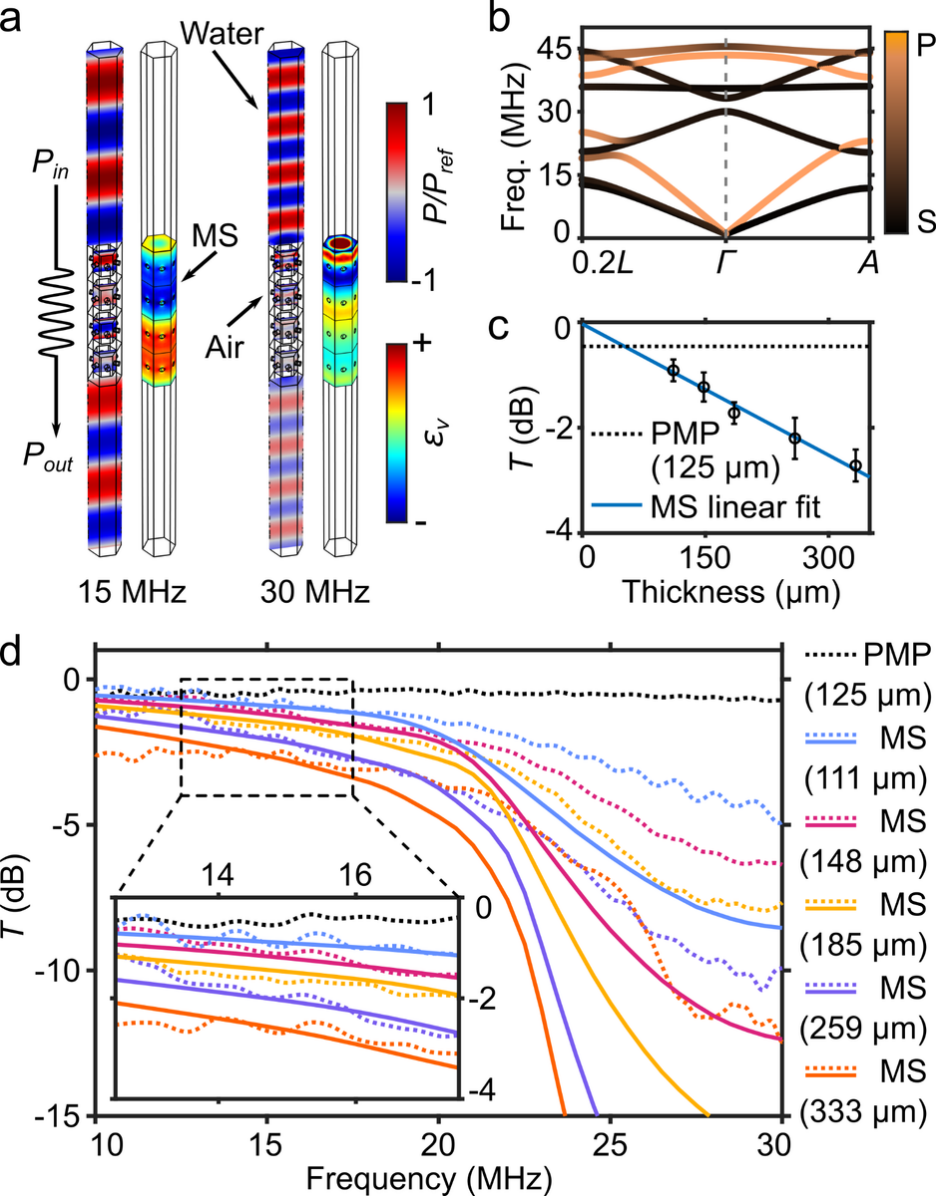
Characterization of dynamic mechanical properties of metaskulls. a, FE model for the ultrasonic wave propagation at 15 and 30 MHz through 4 unit cells of the honeycomb plate-lattices in water, assuming infinite periodicity in the lateral directions. (Left) Pressure distribution within the water surrounding a metaskull and the air inside the cavities. Both water and air pressures are normalised by the peak pressures in each medium. (Right) The volumetric strain distribution within the honeycomb metamaterials. b, The dispersion curves of the honeycomb metamaterials in both **Γ**-A and **Γ**−0.2L directions. Curves’ colours indicate the longitudinal polarisation (see SI) of each mode (blue: pressure mode, red: shear mode, and purple: hybridised mode). The graphical representation of each wavevector in the corresponding Brillouin zone is shown in the SI. c, Experimental transmission coefficients of the MS samples with different thickness (111, 148, 195, 259, and 333 µm) averaged between 13.75 and 17.5 MHz, shown with error bars. The slope of the linear regression plot (blue, solid, r2 = 0.99) is −83.0 dB/cm, whereas the attenuation coefficient of a 125-µm-thick PMP film (black, dashed) is −36.6 dB/cm. d, Experimental (dotted) and numerical (solid) transmission curves of PMP films (black, dotted) and the metaskulls with varying thickness, with respect to frequency. Discrepancies between experimental and numerical results are attributed to the finite size of the experimental samples.

We compute the dispersion curves in the ***Γ***-A direction in the Brillouin zone when excited by plane acoustic waves in the *z*-direction, to investigate the wave propagation characteristics of the honeycomb lattices used in metaskulls (Fig. 3b). The proposed lattice design was selected to have a linear, longitudinal branch around 15 MHz, which allows dispersionless propagation of waves around the fUSI operating frequency. A drawback of acoustic imaging through the skull is the known energy loss in the bone due to the hybridization between the longitudinal and shear modes^43^. We avoid such normal-to-shear coupling by designing the lattice to present a shear mode bandgap between 11.4 and 20.2 MHz, leading to the suppression of shear waves’ conduction through the skull of the subjects^44^. The acoustic impedance of the lattices is matched to biological tissue/water (*Z*_*w*_ = 1.48 MRayl), to reduce reflections from the fluid-metaskull interface. At 15 MHz, the group velocity of the longitudinal waves through the metaskulls’ lattice is *c*_*g*_ = 1,938.7 m/s and the effective density of the lattice is _*eff*_ = 775.6 kg/m^3^, resulting in an acoustic impedance along the vertical direction of Z_MS_= 1.504 MRayl.

We investigate the transmission characteristics of the metaskulls by numerically analysing the frequency dependent response (Fig. 3a). The attenuation through the 4-unit-cell MS at 15 MHz is 1.20 dB, which is significantly smaller than the attenuation at 30 MHz, by 90%. The volumetric strain distribution within the constituent solid as a function of the travelling distance shows that the amplitude of the travelling waves attenuates for both cases, but with much greater loss at the higher frequencies (Fig. S5). The higher transmission loss at 30 MHz is attributed to the presence of a longitudinal band gap between 23.05 and 38.94 MHz, whereas the loss at 15 MHz arises from the viscoelastic dissipation of the polymer itself^45^. With gradually increasing frequency, the slope of the attenuation curve gets steeper and increasingly nonlinear as the frequency surpasses 18 MHz, and the attenuation raises drastically above 20 MHz (Fig. 3c).

We experimentally validate the numerical predictions for the acoustic properties of the metaskulls by measuring the transmission coefficient with respect to the input frequency (Fig. 3d). Microlattice samples with different thickness (111, 148, 185, 259, and 333 µm) are used for the transmission measurements. The discrepancy between the transmission coefficients at higher frequencies measured in experiments and simulations is due to the finite size of the experimental samples and to boundary effects. To compare results, we average and linear-fit the transmission coefficients between 13.75 and 17.5 MHz for each sample, and compare them to the 125-µm-thick PMP film, as a reference (Fig. 3c). The curve-fitted transmission loss data extrapolate to the origin, implying zero reflections at the water-metaskull interface, since the metaskulls are acoustically matched to biological tissue. The attenuation coefficient of the metaskulls, 83.0 dB/cm, is larger compared to that of the PMP film, 36.6 dB/cm, at 15 MHz. We attribute the higher attenuation observed in the metaskull to the presence of interfaces and defects resulting from additive manufacturing. However, we show in the subsequent sections that the fUSI qualities are acceptable even with the slightly increased attenuation.

fUSI is based on the ultrafast transmission of plane acoustic waves in tissues to capture subtle blood flow changes caused by neurovascular coupling. To achieve maximum contrast, coherent compounding of tilted plane-waves was performed^46^. Typically for fUSI, four to ten angles between − 10° to + 10° are used to form a single coherently compounded image. We analysed dispersion curves and transmission properties for varying tilting angles and directions of the incident wave fronts (Fig. S8). The incident angles were tilted in the *xz*-plane based on the Brillouin zone of a hexagonal lattice. Starting from ***Γ***-A, the dispersion curves were computed with a gradually increasing incident angle toward the ***Γ***-L direction (Fig. S8). With greater tilting angle, the longitudinal modes in the lattice get more hybridised with the shear modes, making it less effective at transmitting the acoustic wave energy. We observe that the original dispersion behaviour in ***Γ***-A remains relatively unchanged until ***Γ***−0.2L, which corresponds to a 14.8° tilting. We numerically investigate the transmission performance of the metaskulls as a function of varying incident angle of plane acoustic waves, at 15 MHz (Fig. S9). The transmission curve of the 4-unit-cell MSs is almost flat up to 14.8° with only 0.16 dB reduction from 0°, but the curve shows steep decrease to −2.55 dB at 30° and to −5.81 dB at 40°.

### In vivo transmission characteristics through metaskulls show high SNR

**Figure 4:**
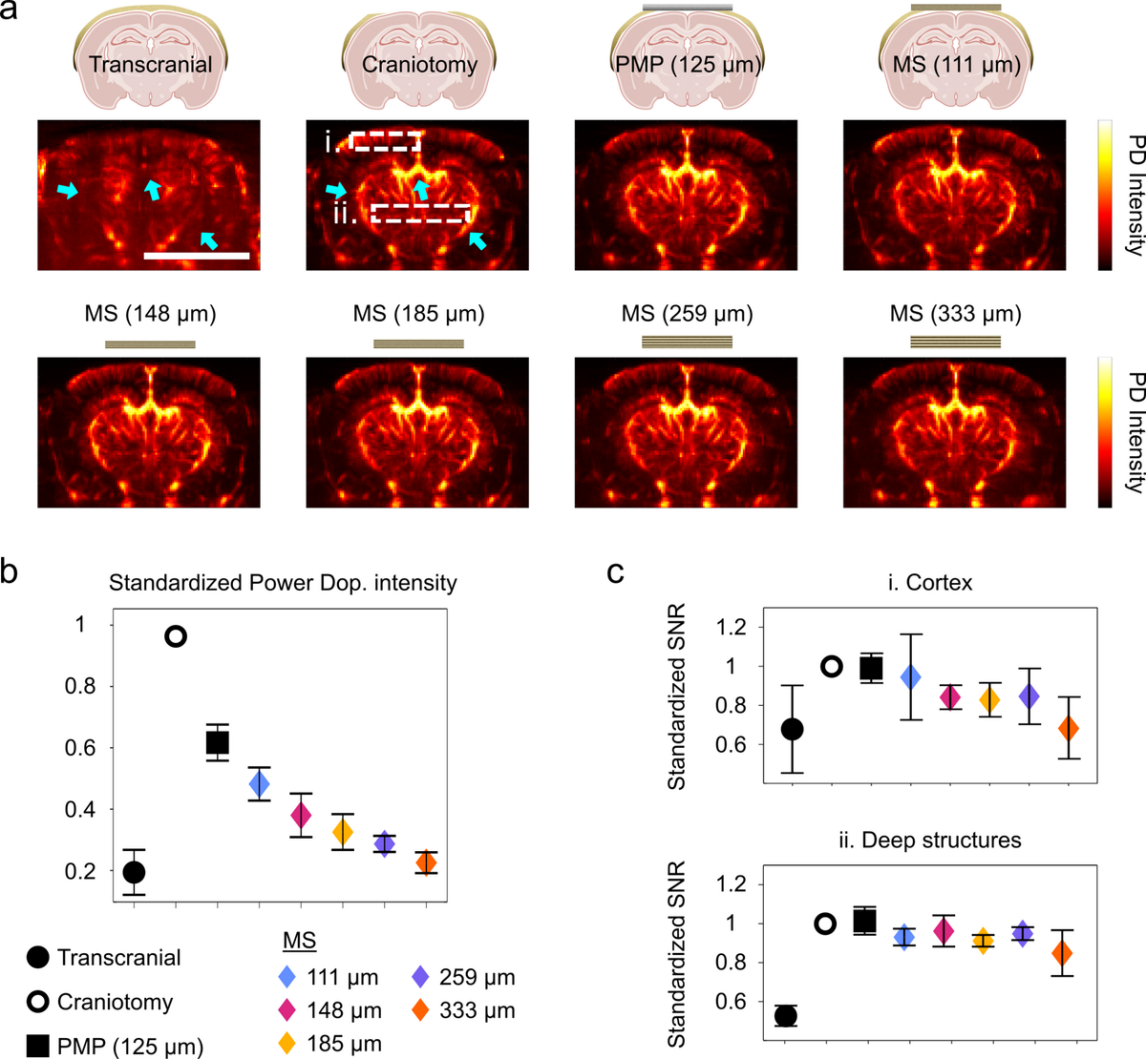
Side-by-side comparison of different skull replacement material for high sensitive cerebral power Doppler imaging in mice (N = 4). a, Cerebral power Doppler imaging of one mouse through the intact skull, after craniotomy without any material, and through a layer of PMP (125 μm) or MSs (111, 148, 185, 259, and 333 μm) (Representative mouse). b, Standardised total intensity of the power Doppler images with respect to the skull replacement material. c, Standardised SNR of the power Doppler in i. the cortex and ii. the deeper structures. Scale bar: 5mm. PD = Power Doppler.

To validate the fitness of the metaskulls as skull prostheses for high sensitivity fUSI, we conduct in vivo imaging studies using a total of 6 mice. We test four mice to compare the SNR performance of the power Doppler images of the brain through two types of cranial windows of varying thickness (Fig. 4), and two for the longitudinal monitoring of fUSI performance after cranial implantation of the metaskulls (Fig. 5).

We first acquire a transcranial cerebral power Doppler image in anaesthetised mice as a reference. We then perform a craniotomy to open a cranial window and to compare the fUSIe performance through different skull replacement materials. The metaskulls with varying thickness and a PMP film (125 µm) are positioned on top of the brain to cover the cranial opening. The thickness of the metaskull samples are 111, 148, 185, 259, and 333 µm, which correspond to 3, 4, 5, 7, and 9 layers, respectively, along the thickness direction. The size of the metaskull windows is 1 cm x 0.25 cm, consisting of ~ 75,000 honeycomb unit cells per layer. All power Doppler images are acquired on the same coronal plane for all conditions (Bregma − 2.5 mm).

The standardised intensity maps obtained from the power Doppler using different types of windows are plotted side by side (Fig. 4a). We observe strong attenuation of the transcranial power Doppler signal relative to the image acquired after craniotomy. The main arteries (see blue arrows) are indiscernible, also showing poor in-depth signal. Without any covering after craniotomy, cortical vessels are clearly visible, as well as the deeper vessels in the thalamic regions (blue arrows). We then cover the brain with a PMP film or the metaskulls with different thickness, and no clear structural change from the acquired images was noticeable. The average intensity in the power Doppler images is the highest in the case of craniotomy and the lowest in the transcranial case (Fig. 4b). Taking the intensity from the craniotomy as a reference, the PMP case recovered 60% of the reference intensity, while the metaskulls showed gradual decrease from 50% (111 µm) to 20% (333 µm) of the reference intensity with increasing thickness.

We quantitatively assess the signal sensitivity of the ultrasound images through the metaskulls by analysing the SNR of the acquired blood vessel mappings. The normalised SNR in cortical regions and in deeper structures (defined by the white-dotted squares in Fig. 4a) are shown for all the cases (transcranial, craniotomy, PMP, and metaskulls) (Fig. 4c). The transcranial SNR is evaluated as a reference, and one can again observe a major loss in SNR in the transcranial case (70% of the craniotomy signal in the cortical region, and 45% in the deeper structures). The SNR obtained after covering the brain with a PMP window is almost 95% of the craniotomy SNR, both in the cortical and deeper regions. Similarly, the metaskulls only cause a slight decrease in SNR for the thinner case (92% at 111 µm) for both cortical and deeper structures, while the SNR gradually decreases to 70% as the thickness of the metaskull increases to 333 µm.

We show that the metaskulls induce more than 50% decrease in intensity (this decrease is around 40% for the PMP material) compared to the craniotomy case in the power Doppler images, but that the SNR remained above 80% of the craniotomy SNR.

### Longitudinal study confirms biocompatibility and functional signal conservation over time

We surgically implant the metaskulls in mice (*N* = 2) to evaluate the biocompatibility of the constituent polymer, and conservation of the functional signal through the implant over a long period of time is observed (> 4 months). Power Doppler scans are performed at days 10, 20, 34, 82 and 120 after surgery, during which we stimulate the visual system to measure evoked activation in the lateral geniculate nuclei (LGN).

At day 10 after the implantation, the power Doppler scan allows visualisation of the brain vessels with great sensitivity from the cortex all the way down to the amygdala. Visually evoked response is clearly visible as the two LGN were activated, which led to a higher volume of blood to flow. Throughout the study, the degradation of the power Doppler signal is observed, resulting in a shallower distinction of the blood vessels in the deeper structures (see Fig. 4 for definition of the deep structures). However, the activation of both LGN is distinguishable throughout the whole functional study from day 10 to day 120 with more than 50% correlation with the stimulation pattern.

### Advantages, limitations, and outlook

In this work, we implanted acoustic metamaterials into living animals for ultrasound imaging and brain protection. The proposed metamaterial cranial window, or “metaskull”, allows for fUSI in small animal brains with long-term stability and lasting signal sensitivity, over 120 days. The metamaterials’ inner geometry was architectured so that the metaskulls can accomplish minimally attenuative transmission of ultrasonic waves at 15 MHz. The high stiffness of the designed microlattices offers robust protection to the brain. Our approach surpasses other widely used solutions for cranial windows, e.g., PMP, since the complex shapes and curvatures of the skull can be easily tailored via 2-photon lithography.

The transmission characteristics of the metaskulls could be improved by exploring alternative manufacturing approaches. For example, the viscoelastic nature of the constitutive polymer, IP-S, and the interfacial friction between building blocks cause undesired signal attenuation. To address this issue in future studies, the polymer could be carbonised through pyrolysis to create a more brittle structure^47^.

Further research should aim at incorporating additional functionalities into the metaskulls, to adapt its functionalities to other biomedical applications. For instance, the metaskulls could be designed as a focusing lens and/or spatial sound modulator, to manipulate ultrasound waves on demand^48^. The use of metaskulls could also achieve safer photoacoustic imaging, which requires the transmission of ultrasound generated through photonic stimulation^49^. Additionally, the scalability of the metaskulls has a potential to create chronic human skull prostheses, enabling long-term monitoring, treatment, and stimulation using ultrasound^50^.

## Methods

### FEA for the mechanical characterizations

We performed the numerical mechanical characterization of a metaskull under quasi-static or dynamic loading via FE analysis (Fig. 2b, 3a). The models used for the simulations consist of a 4-unit-cell thick metaskull with a 300 µm diameter circular face. Mimicking the compression experiment setup shown in Figure 2a, 50 µm X 50 µm square-faced punch was compressed against the plate-lattice domain. Assuming linear elasticity and geometric linearity, we computed the simulation to plot the von Mises stress distribution on *xz*- and *yz*-planes, showing the stress concentration along the drainage holes. In addition, we built the transmission models to assess the acoustic characteristics of the travelling pressure waves with respect to the frequency and the angle of incidence (Fig. 3a).

### Calculation and visualisation of the dispersion curves

Numerical simulations were performed using a commercial FE software (COMSOL Multiphysics^®^). The dispersion curves of a metaskull were derived by numerically solving the characteristic equation of the honeycomb unit cell. Bloch-Floquet periodic boundary conditions were applied on all sides, assuming infinite periodicity. With evenly-spaced wavenumbers sweeping within the irreducible Brillouin zone, the eigenfrequencies below 50 MHz were calculated to best represent the behaviour of the metamaterials around the operating frequency of fUSI for small animals (~ 15 MHz). With incident plane waves travelling in the *z*-direction, ***Γ-A***, the volume-averaged displacements in each direction were normalised with the total volume-averaged displacement. We determined the longitudinal polarisation of each normal mode based on the dominant direction of deformation. The longitudinal polarisation factor is defined as where,, and are the displacements in *x*, *y*, and *z* directions, respectively. If the longitudinal polarisation factor of one normal mode is close to 1, the mode has dominant pressure wave behaviour, as opposed to when the transverse mode is dominant and the polarisation factor is close to 0. With blue being purely longitudinal and red being purely transverse, the dispersion curves were plotted to indicate the polarisation of the mode at each solution (Fig. 3b). The dispersion curves of the honeycomb unit cell in different wave directions were calculated by sweeping the Brillouin zone in the reciprocal domain. The wavenumber vectors *k* = (*k*_*x*_, *k*_*y*_, *k*_*z*_) parallel to ***Γ-A***, ***Γ-αL***, ***Γ-αH*** with *α* = 0.2, 0.5, or 1, are used for the dispersion curves.

### Transmission simulation

The input and output pressure field, padded with perfectly matched layers, sandwich a column of honeycomb plate-lattices composed of a finite number of unit cells (*n* = 3, 4, 5, 7, and 9). We imposed the Bloch-Floquet periodic boundary conditions on the side faces to assume infinite periodicity in the lateral directions. The viscoelastic dissipation of the constituent polymer was incorporated by feeding an isotropic structural loss factor (*η* = 0.075) to the materialistic model^45^. We evaluated the amplitude of the travelling pressure waves in the output pressure field, and showed the transmission curves in dB, (Fig. 3d).

### Micro fabrication process of metaskulls

We adopted the microscale 2PP technique for the fabrication of the metaskulls with intricate inner structures (Nanoscribe GmbH & Co. KG). The photoresist, IP-S, in the form of highly viscous liquid becomes acrylic when cured under laser irradiation. After printing the desired MS geometry, we develop the MS using propylene glycol monomethyl ether acetate (PGMEA) and isopropyl alcohol (IPA). The presence of the drainage holes allows the remaining photoresist to be thoroughly removed from the cavities. We measured the mass of the MS samples before and after underwater tests to confirm that the cavities are saturated with air, trapped inside the structure due to surface tension. For microscopic images, only 25 X 24 X 4 arrays of unit cells were printed, which resulted in a sample with outer dimensions of 590 µm X 574 µm X 148 µm (Fig. 2a). Since the single printing size of the Nanoscribe Photonic Professional GT (300 µm X 300 µm X 300 µm) is smaller than the final dimension, smaller blocks were stitched together to form a larger final structure.

### Compression experiments on metaskull samples

The quasi-static characterization was performed using a micromechanical testing tool (FemtoTools AG, FT-MTA02) (Fig. 2a). A displacement-controlled probe tip with a square end (FT-S100,000) was compressed against a metaskull sample composed of honeycomb unit cells for force measurement. The size of the sample used for testing (700 µm X 700 µm X 148 µm) is larger than the front end of the tip (50 µm X 50 µm), so that boundary effects could be neglected (Fig. S2). The probe tip recorded the force signal as the measuring arm travelled downward 2 µm from the top surface. Calibration test against a rigid substrate surface was done prior to the measurements to offset the deformation of the measuring arm under given force. The reference stiffness of the measuring system, *K*_*ref*_ = 28,000 N/m, was then used for the calibration. We took the sample’s effective stiffness from the beginning of the unloading curve, which indicates the global response of the lattice.

### Acoustic transmission experiments

We used zero-padded, Hann-windowed single-cycle sinusoidal bursts as input waves for the underwater transmission tests. The signals centred at multiple different frequencies were sent through the metaskull samples using an immersion transducer (Olympus, V356-SU). The output signals were recorded by a hydrophone (Precision Acoustics, 0.2 mm needle) and Fourier-transformed for the evaluation in the frequency domain. We plotted the transmission coefficients of the metaskulls with varying thickness (111, 148, 175, 259, and 333 µm) and a PMP film (125 µm) in Fig. 3b.

### Animal surgeries for in vivo experiments

All animal experiments were conducted under protocols approved by the Institutional Animal Care and Use Committee of the California Institute of Technology. The in vivo experiments presented were performed on C57BL/6J mice (Jackson Laboratory) aged between 6 to 8 weeks. No randomization or blinding were necessary in this study.

### Side-by-side comparison of different skull replacement material

Four mice were used for the cranial window characterization study. Mice were anaesthetised with 2–3% isoflurane with their heads fixed on a stereotaxic frame. After the incision and stabilisation of the skin, the skull was exposed and rinsed with sterile saline. Ultrasound coupling gel is applied on top of the skull, then we acquired a first transcranial power Doppler image set (“Transcranial” case in Figure 4a) following the parameters described in the fUSI acquisition section. A skull window (1 cm x 0.4 cm) was then removed by drilling (Foredom) at low speed using a micro drill steel burr (Burr number 19007–07, Fine Science Tools). Care was taken not to damage the dura and to prevent inflammatory processes in the brain. We acquired a second transcranial power Doppler image set where only ultrasound coupling gel is applied on top of the brain (“Craniotomy” case in Figure 4a). Then, successive implant sheets for PMP and metalskull cases were positioned on top of the brain for additional power Doppler acquisitions.

### Surgical implantation of honeycomb lattices for chronic imaging of the brain in mice

Two mice were implanted with the metaskull and used for the longitudinal study. Mice were anaesthetised with 2%–3% isoflurane, with their heads fixed on a stereotaxic frame. After the incision and stabilisation of the skin, we removed a rectangular skull window (1 cm x 0.4 cm) by drilling at low speed using a micro drill steel burr. The window corresponds to the coronal planes from Bregma −2 mm to Bregma −2.5 mm. Care was taken not to damage the dura and to prevent inflammatory processes in the brain. We dropped 20 µl of artificial dura on top of the exposed brain to provide an aqueous layer between the brain tissue and the implant. A 1 cm x 0.5 cm metaskull window was sealed in place with the layer of acrylic resin.The surgical procedure took 45 min to 1 h. Animals recovered quickly, and after a conservative 10 days resting period, they were used for the data acquisition via fUSI.

### fUSI acquisition

fUSI visualises neural activity by mapping local changes in cerebral blood volume (CBV). CBV variations are tightly linked to neuronal activity through the neurovascular coupling and are evaluated by calculating power Doppler variations in the brain. fUSI was performed using a 15 MHz ultrasonic probe (L22-14vX, 15 MHz, 64 elements, 0.11 mm pitch, Verasonics) connected to a Verasonics Vantage ultrasound system (Verasonics) driven by custom MATLAB (MathWorks) transmission scripts. Each power Doppler image was obtained from the temporal integration of 300 compounded frames acquired at 500 Hz frame rate, using 5 tilted plane waves separated by 3° (−6°, −3°, 0°, 3°, and 6°) acquired at a 2,500 Hz pulse repetition frequency. Power Doppler images were then repeated every second (1 Hz image framerate). Each block of 300 images was processed using a SVD clutter filter to separate tissue signal from blood signal to obtain a final power Doppler image exhibiting CBV in the whole imaging plane.

### Functional activation of mice visual system

To evaluate the sensitivity of fUSI through metaskull long-term, we stimulated the visual system of the metaskull-implanted mice over multiple days (day 10, 20, 34, …). We delivered visual stimuli using a blue LED positioned at 3 cm in front of the eyes of the mice. Stimulation runs consisted of periodic flickering of the blue LED using the following parameters: 30 s of rest followed by 30 s of a flicker.

### Activation maps

Correlation maps were computed individually from the normalised correlation between each pixel’s temporal signal with the visual stimulus patterns (Pearson’s product moment) using MATLAB (MathWorks).

### SNR calculation

For the SNR performance through different materials and thicknesses study (Fig/ 4), we delimited two regions: one region in the cortex (region i.) and one region in the deeper structures (region ii.). Each region was of dimensions: 10 × 5 (lateral x depth) pixels. For each depth, we measured the intensity profile and calculated the local minima and maxima along the lateral axis (Fig. S10). SNR was calculated as:

$$SNR=\frac{\sum_{depth=1}^{5}local\;maxima}{\sum_{depth=1}^{5}local\;minima}$$

## Supplementary Material

Supplement 1

## Figures and Tables

**Figure 1:** Design and structure of a metaskull. a, Concept schematic illustrating ultrasound brain imaging through a metaskull. A 3D model of the inner structures of the metamaterials shows a periodic tessellation of honeycomb unit cells, with h = 37 µm, w = 32.3 µm, th = 7.5 µm, and tw = 4.5 µm. b, The metaskulls can fit an arbitrarily shaped region in a curved parietal lobe of the mouse skull (dashed blue region). c, The metaskulls are fabricated with a microscale 2-photon polymerization technique. SEM images of a metaskull’s sections, from the top and isometric views. Scale bars: b, 1 cm; c, 200 µm (left), 100 µm (bottom right), and 20 µm (top right).

**Figure 5:** Longitudinal fUSI study in mice after metaskull implantation. a, Longitudinal study protocol: mice are implanted with a metaskull (148 µm) at day 0. They are then examined at day 10, 20, 34, 82 and 120, during which visually evoked activity is recorded. b, Power Doppler images are acquired around coronal plane B-2.2 mm showing the structure of the vascular network (top, hot colours) and the activated LGN following visual stimulation (bottom, cold colours)
