# Supplementary material for: Microscale acoustic metamaterials as conformal sonotransparent skull prostheses
Source: Res Sq. 2023 May 10:rs.3.rs-2743580. Preprint. [Version 1] doi: 10.21203/rs.3.rs-2743580/v1 (PMC10197820; doi:10.21203/rs.3.rs-2743580/v1)
Supplement: Supplement 1 [file NIHPPRS2743580V1-supplement-1.pdf]

Supplementary Files

This is a list of supplementary files associated with this preprint. Click to download.

- Microscaleacousticmetamaterialsasconformalsonotransparentskullprosthesessupplementary.docx
